# Predicting carer health effects for use in economic evaluation

**DOI:** 10.1371/journal.pone.0184886

**Published:** 2017-09-26

**Authors:** Hareth Al-Janabi, Andrea Manca, Joanna Coast

**Affiliations:** 1 Health Economics Unit, Institute of Applied Health Research, University of Birmingham, Birmingham, United Kingdom; 2 Centre for Health Economics, University of York, York, United Kingdom; 3 Department of Population Health, Luxembourg Institute of Health, Luxembourg City, Luxembourg; 4 School of Social and Community Medicine, University of Bristol, Bristol, United Kingdom; 5 The National Institute for Health Research Collaboration for Leadership in Applied Health Research and Care West (NIHR CLAHRC West) at University Hospitals Bristol NHS Foundation Trust, Bristol, United Kingdom; University of Exeter, UNITED KINGDOM

## Abstract

**Background:**

Illnesses and interventions can affect the health status of family carers in addition to patients. However economic evaluation studies rarely incorporate data on health status of carers.

**Objectives:**

We investigated whether changes in carer health status could be ‘predicted’ from the health data of those they provide care to (patients), as a means of incorporating carer outcomes in economic evaluation.

**Methods:**

We used a case study of the family impact of meningitis, with 497 carer-patient dyads surveyed at two points. We used regression models to analyse changes in carers’ health status, to derive predictive algorithms based on variables relating to the patient. We evaluated the predictive accuracy of different models using standard model fit criteria.

**Results:**

It was feasible to estimate models to predict changes in carers’ health status. However, the predictions generated in an external testing sample were poorly correlated with the observed changes in individual carers’ health status. When aggregated, predictions provided some indication of the observed health changes for *groups* of carers.

**Conclusions:**

At present, a ‘one-size-fits-all’ predictive model of carer outcomes does not appear possible and further research aimed to identify predictors of carer’s health status from (readily available) patient data is recommended. In the meanwhile, it may be better to encourage the targeted collection of carer data in primary research to enable carer outcomes to be better reflected in economic evaluation.

## Introduction

It is increasingly recognised that the health benefit that family carers may derive from healthcare interventions is a relevant consideration in economic evaluation, whether one takes a societal or healthcare perspective [1,2]. This is underscored by recent guidance from the 2^nd^ US Panel on cost-effectiveness which advocates *“the reference case cost-effectiveness analysis [should include] QALYs accruing to patients and to any other affected parties such as caregivers”* ([3], p.1097). There is also a policy imperative to this, given the increasing reliance European health systems have on family care. One way in which carers’ health and wellbeing can ultimately be maintained is by considering these outcomes at the outset when evaluating health and social care interventions [4].

Obtaining data on carers’ outcomes in addition to patients’ outcomes as part of a clinical trial, or an observational study, is an obvious way of building carer outcomes into economic evaluation. In practice, though, this may not always be feasible. One option might be to ‘predict’ carer outcomes based on other data collected in a primary research study, for example patient outcomes. In this study, we set out to assess whether changes in carers’ health status could be predicted from data relating to the patient’s health. The study was conducted in the UK, using the chronic after-effects of meningitis, as the clinical case study. Vaccinating against meningitis may yield health benefit, not just for patients, but also for the wider family network, if vaccination reduces long-term morbidity from meningitis and the associated caring responsibilities of family members [5]. Our specific objectives were:

1. To examine whether it was feasible to predict changes in health status for carers at the *individual* level using patient health and demographic data; and
2. To examine predictions for subgroups of carers, given that economic evaluation is based on the mean health effects falling on a group of the population

In the following section we review the relevance of carer outcomes in economic evaluation, the potential for predicting carer health status from patient health data, and outline a conceptual framework for study. We then describe our methods to collect data, estimate predictive models, and test their predictive accuracy. The results and implications are discussed in the final two sections.

## Background

### Informal care in economic evaluation

In an economic evaluation, informal care can be considered on both the input and outcome side [6–8]. On the input side, the time devoted to caregiving represents a ‘resource’ that can be used up [9,10]. On the outcomes side, a carer’s wellbeing can be affected by both the process of informal care and the distress of seeing a loved one in poor health [7,11,12]. Economic evaluations that take a wide societal perspective ought to consider both the costs and outcomes associated with informal care [1], whilst paying attention to the potential for double counting [7,13,14]. If a healthcare perspective is taken in the economic evaluation of health technologies, then carers’ time cannot be considered in the analysis as the costs are borne by agents outside the healthcare system; however, under the healthcare perspective carers’ health outcomes remain relevant [1,15–17].

One approach to valuing carers’ quality of life outcomes is to use quality of life tools to measure the carers’ quality of life and ‘off-the-shelf’ social tariffs to value changes in quality of life [18]. Quality of life tools include those capturing *health*-related quality of life [15,19], *care*-related quality of life [20,21] and capabilities [22–24]. An advantage of using health-related quality of life measures is that carer and patient outcomes are measured using the same numeraire and estimation of the total health impact of the disease/technology can be more easily derived. Additionally clinical studies may measure carer burden or quality of life using a non-preference-based measure [25]. These outcomes may potentially still be considered in an economic evaluation as part of a cost-consequence analysis [26,27].

A second approach is to value carers’ outcomes directly. This can be done using time trade-off or standard gamble techniques to estimate how much of their own life a carer would be willing to sacrifice to improve a patient’s health state [28,29]. Alternatively, carers can be asked to express how much they would be willing to accept to undertake more caring tasks [30]. This provides a monetary valuation of the impact of caring that (in theory) incorporates the health effects of caring.

In this study, we focus on the health-related quality of life approach to valuing carer outcomes, as this is consistent with most guidance for applied economic evaluation [16]. For example, recent NICE guidance, which takes a healthcare perspective, suggests that analysts ought to comprise all direct health effects, including those falling on carers [31]. Similarly, where a societal perspective for economic evaluation has been advocated, for example in the Netherlands or the United States, the inclusion of carer health has also been recommended [1,3].

Reviews of applied economic evaluations show that carer outcomes are rarely considered in applied economic evaluations [32], even for conditions associated with substantial informal care burden [2]. This is of concern for two reasons; first if included, carer outcomes would be expected to alter the incremental cost-effectiveness ratio and in some cases this may affect technology adoption recommendations [2,15,33]; second, neglecting carer outcomes overlooks important equity implications of funding decisions. As data on patient outcomes will invariably be available when evaluating the cost-effectiveness of an intervention, it seems pertinent to explore whether this can be used to predict outcomes for carers, to enable the consideration of both outcomes within an economic evaluation.

### The relationship between carer and patient outcomes

Any predictive modelling of carer health from patient data assumes an underlying association between the outcomes of carers and patients. A theoretical basis for anticipating that patient and carer outcomes are likely to be intertwined comes from psychological models of stress adapted to the caregiving context [34]. Caring for someone in poor health is a potentially stressful experience that may result in a range of adverse outcomes, including adverse health outcomes, for the carer. Stress is likely to arise from ‘primary’ factors such as the patient’s dependency and ‘secondary’ factors resulting from that dependency, such as family conflicts [34].

It has also been noted that the combined effects of prolonged distress, the physical demands of caregiving, and the biological vulnerability of some carers may affect carers’ physical health [35]. Carers’ health may also suffer if they can no longer undertake joint physical activities together with the patient, such as exercise or leisure. The psycho-social nature of caring means that the most significant effects of caring will often be in terms of the carers’ mental health [36–39]. However, the psychological impact of caring will vary from carer to carer and there is evidence of positive aspects of caring, such as fulfilment and relationship benefits for many carers [40–42].

A number of studies reveal that patient disability adversely affects carer health across a wide range of clinical conditions, including childhood illness [43] and neurological conditions [44]. Econometric modelling of carer wellbeing suggests carers suffer from worse wellbeing than non-carers, and that carer wellbeing is progressively lower as the recipient’s care needs increase [45]. However, the relationship between carer health and patient outcomes is not a simple one and is unlikely to be consistent across conditions. [18] p.496 suggest: “*…most of the literature reported small negative effects [of patient illness on the carer]… likely correlated with the condition*, *population affected*, *as well as measurement technique…*”.

**Fig 1:**
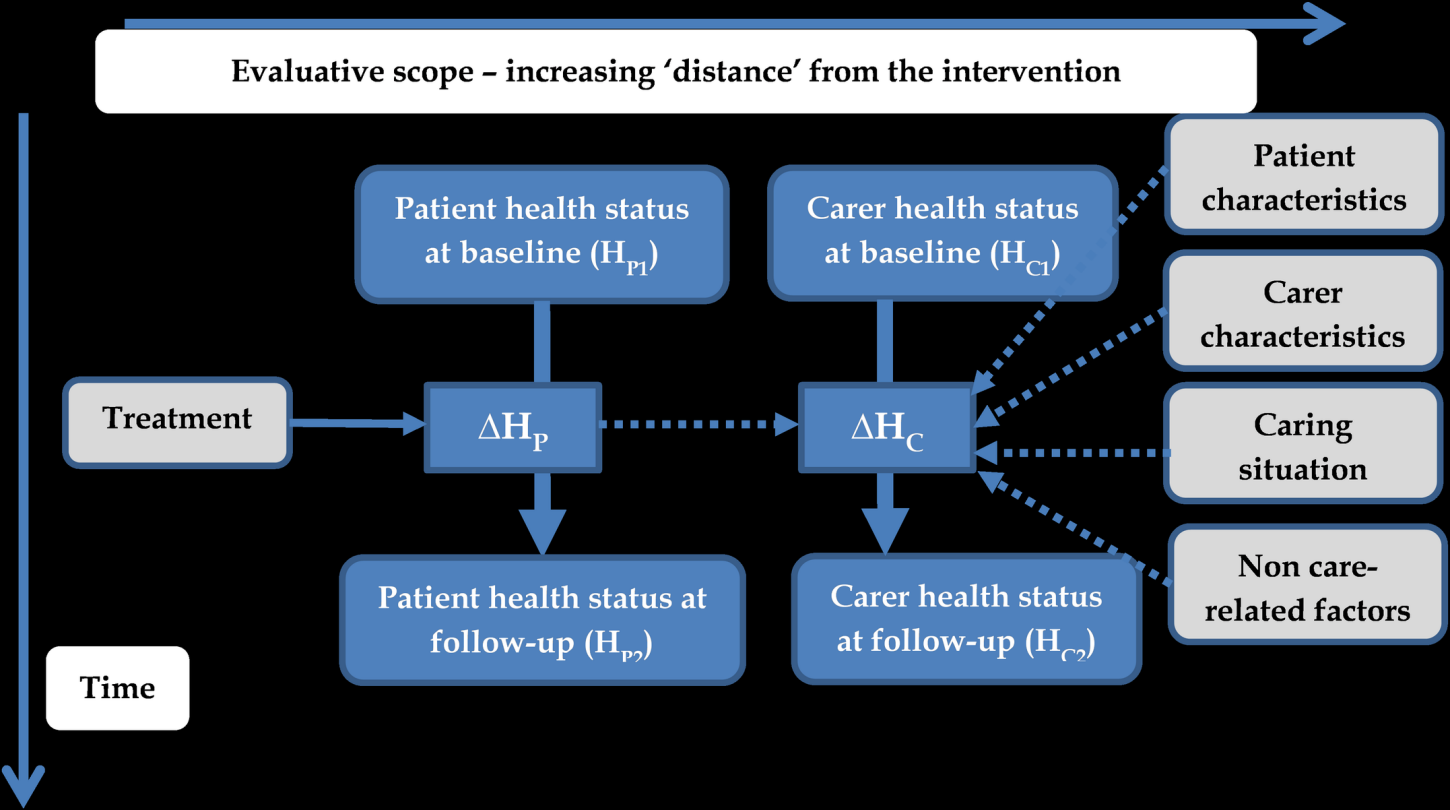
Conceptual framework for predicting health effects of treatments on carers. A causal relationship between patient and carer outcomes is hypothesised, based on what we know about the experience of informal care. However, for the purposes of the modelling, we assume only that there is an association between the two outcomes. Where we anticipate an association (for example between the treatment and ΔH_P,_ or between the caring situation and ΔH_C_) we have used dashed lines.

A conceptual framework for predicting changes in carers’ health status (ΔH_C_) is laid out in Fig 1. This postulates that ΔH_C_ could be associated with a new patient treatment if there is an underlying relationship between the change in carer health status and patient health status (ΔH_P_). In estimating the total effects of the intervention one should in theory consider ΔH_P_ and ΔH_C_. The latter could be directly calculated by measuring a carer’s health status before and after an intervention. Alternatively ΔH_C_ could be directly predicted from ΔH_P_ and other variables that are likely to be related to ΔH_C_ such as those listed on the right-hand side of the figure. Table 1 summarises some of the likely determinants of ΔH_C_. Of course there are multiple determinants of ΔH_C_ and only some of these, such as patient health and certain demographic characteristics, may be easily obtained from a particular study.

**Table 1:** Potential determinants of carer health status.

	Nature of relationship
**Patient health status**	Greater patient impairment is associated with poorer carer health status [12,18]. Improvements in any aspect of the patient’s health status would be anticipated to have a positive impact on carer health status, given the potential for reduced informal care needs and anxiety over the patient’s condition.
**Patient characteristics**	There is some evidence that suggests caring for older patients and/or male patients is associated with higher carer burden [25]. Thus changes in carer health status may be related to the patient’s age and sex.
**Patient healthcare use**	If the patient receives formal healthcare this may relieve some of the burden on the carer, benefitting the carer’s health status. However, the receipt of formal healthcare may signify a serious and stressful episode for the patient and therefore be associated with negative impacts on the carer’s life.
**Caring situation**	Specific features of the caring situation such as the nature of the caring relationship, the amount of care provided, and the type of tasks undertaken may impact on the carer’s health status [35,39]. In general increases in caring responsibilities and co-residential care to a close relation would be expected to adversely affect carer health status.
**Non-care-related factors**	The health status of carers, as with other member of the population, will also be affected by their own age, sex, genes and environment [36,46].

## Methods

This research is based partly on data collected for an earlier study of the family impact of meningitis [5]. For the purpose of the present study we followed-up participants to the survey, measuring their health status and the health status of patients (i.e. survivors of meningitis) at baseline and after 12 months. We used these data to examine whether changes in carers’ health status could be predicted using changes in patients’ health status. Details of the earlier study, further data collection, and estimation and testing of predictive models are reported below. The study protocol was approved by the University of Birmingham's Life and Health Sciences Ethical Review Committee (ERN_11–0191).

### Family impact of meningitis case study

Meningitis is a potentially deadly condition, but most patients survive the disease. Of those who do survive, a sizeable minority (around 20–30%) will develop long-term physical or mental health problems, such as learning difficulties, hearing and sight loss, and balance problems [47,48]. Meningitis is most prevalent amongst the very young, so any after-effects are likely to affect family carers of the survivors of meningitis. Because meningitis is associated with a range of long-term health problems, but is potentially preventable, it provides an important case study in understanding the nature of spillover impacts on carers.

The baseline data for this study come from a UK-wide survey of families affected by meningitis. The study was conducted in May 2012, and data were gathered on the health status (and many other outcomes) of 1,218 survivors of meningitis (‘patients’) and 1,587 family members. Most family members were parents of the patients. Given the young age and high level of disability of many patients, data about the patient was proxy-reported by the family members. Recent analysis of these data suggested proxy-reporting did not introduce any systematic bias to the association between family member and patient health status [5]. The earlier study focused on measuring the impact of meningitis on the health status of family members. The findings of this previous study have been used in decision-modelling that showed that the cost-effectiveness of MenB vaccination was sensitive to a number of factors, including the assumption made about the scale of family impact [49]. This re-evaluation of cost-effectiveness also influenced the UK advisory body on vaccination who subsequently recommended vaccination [49,50]. In the present study we are interested in the methodological question of whether carer health effects (ΔH_C_) could be ‘predicted’ from data relating to the patient.

### Further data collection for predictive modelling

We collected data at two time points which enabled us to observe and predict changes in health status for individual carers. Previous studies have adopted cross sectional designs focused on predicting outcomes at a single point in time [51,52]. Collecting data at two time points however provides a more direct, and potentially valuable, test of predictive ability of models as it is ultimately changes in health status (rather than the health status per se) that are used to estimate benefits within economic evaluation.

In May 2013, we sent a 14-page follow-up questionnaire to all family members who returned a questionnaire in 2012 or were involved in the pilot study (n = 1,627). The format for the follow-up questionnaire was very similar to the baseline questionnaire, which had been piloted with a focus group (n = 6) and a sample of family members (n = 30). The follow-up questionnaire was completed by family members and included sections on:

- The health status of the patient
- The after-effects of meningitis
- The social network of the patient
- Informal care provided by the family member
- The health status and wellbeing of family member
- Perceptions of how meningitis had affected the family over the last year.

Our particular focus in this study is on the health status data. For both family members and patients, health status was measured using the EQ-5D-5L [53]. This is a preference-based generic measure of health related quality of life, widely used to measure population health [54] and to inform value for money assessments carried out for a number of funding bodies, e.g. NICE, SMC, CADTH, PBAC, CVZ [55] The EQ-5D-5L is built around 5 dimensions of health (mobility, self-care, usual activities, pain and discomfort, and anxiety and depression), each with 5 possible levels of severity (no problems, mild problems, moderate problems, severe problems, and extreme problems). To estimate health status on a 0 (death) to 1 (full health) scale, we scored responses to the EQ-5D-5L questionnaire using interim value sets for the UK [56].

We included questionnaires in a survey pack with information about the study and a pre-paid reply envelope and posted these to 1,627 family members in the sample frame. Reminder postcards were sent to all members after one week and reminder letters to all non-responders after four weeks. We excluded respondents if the patient had died, the family member had become estranged from the patient, a different family member responded to the follow-up questionnaire, the family member switched focus to a different patient at follow-up, or the patients themselves completed the survey questionnaire. We entered data into a secure database, with 5% of questionnaires being double entered to verify the accuracy of data entry.

### Estimating predictive models of changes in carers’ health status

In order to predict ΔH_C_ for ‘carers’ (as opposed to family members more broadly) we restricted the sample in two ways. First, we excluded family networks where the patient did not develop after-effects from meningitis. Second, we included only one respondent from each of the family networks. In cases where family networks contained two respondents, data on co-residence, social contact, relationship, employment status and gender were used to determine which family member was likely to be closest to the respondent [5]. This process generated a sample of unique carer-patient dyads, where all patients had some degree of health problem.

We then split carer-patient dyads into two equal-sized random samples–a training sample and a testing sample. Other methods exist for checking predictive accuracy of models; these would have allowed us to retain the full sample for estimating the model. Methods include (i) external validation with a different dataset; and (ii) internal validation. Because no external dataset was present, splitting the sample provides a way of conducting internal validation and checking whether the model predictions are generalisable beyond the dataset used to develop them. We used the training sample to estimate the predictive models. The testing sample provided an external sample for testing the predictive accuracy of the models. We used the training sample to predict ΔH_C_, assessed using the EQ-5D-5L, between baseline and follow-up (i.e. over 12 months). The ΔH_C_ variable provides a summary measure of carer health, which could be utilised within an economic evaluation.

We estimated four predictive models of ΔH_C_:

- Model 1: This model predicted ΔH_C_ as function of change in each of the five individual items of the patient health status (described using the EQ-5D-5L). The intuition for this model is based on cross-sectional data analyses indicating an association between the health status scores of carers and patients [12,43,57]. This model is based on evidence suggesting that some aspects of patient health may affect carer health status more than others [57,58]. To assess the change in each item of the patient’s EQ-5D-5L, we generated five categorical variables to indicate whether there had been an improvement, a decline, or no change in each item.
- Model 2: This model predicted Δ H_C_ as a function of any changes in the disabling after-effects of meningitis for the patient (in addition to the variables included in model 1). The purpose of this model was to examine whether ‘clinical’ data on the patient in addition to their health status might better predict Δ H_C_. Information was collected in the survey (at baseline) about the presence or absence of specified after-effects of meningitis [5]. Information was then collected in the follow-up survey on whether any of the specified after-effects had improved or worsened. We used dummy variables to represent the presence or absence of any of the five most commonly reported after-effects of meningitis. There were behavioural/emotional problems, mild learning disabilities, scarring, balance problems, and speech and language problems. We also explored the use of categorical variables representing (carer-perceived) improvements or worsening in each of the five most commonly reported after-effects.
- Model 3: This model predicted ΔH_C_ as a function of patient healthcare use (in addition to the variables included in model 2). We used this model to examine whether patient healthcare use–which may provide additional information on patients care needs–aided the prediction of ΔH_C_. We used three categorical variables to indicate whether, over the last 12 months, the patient used GP services, hospital services as an outpatient, and hospital services as an inpatient.
- Model 4: This model predicted ΔH_C_ as a function of characteristics of the informal carer (in addition to the variables included in model 3). We included variables relating to the carers’ age, sex, relation to the patient, co-residence, and provision of daily care. Whilst the purpose of this study is to determine whether patient data can be used to predict ΔH_C_, we used this model to verify whether carer-specific information is needed to accurately predict carer outcomes.

In all models we additionally controlled for the patient’s age and sex, as these data are likely to be collected routinely in clinical studies. We estimated all models using OLS regression, as ΔH_C_ was approximately normally distributed in this sample. We also investigated two alternative modelling strategies. We first used an adjusted limited dependent variable mixture model. This is recommended as a method for dealing with non-unimodal distributions and gaps in values [59]. We also used a Beta regression model. This is recommended as a method for dealing with the truncated range of values of health-related quality of life data [60]. Comparisons between the estimation strategies revealed very little difference in terms of the p-values produced for individual variables, with OLS performing slightly better across all models on the basis of AIC ad BIC statistics. The comparisons between estimation techniques are shown in the supporting information (S1 File).

Following OLS estimation of the four models, we used each model to predict changes in carers’ EQ-5D-5L scores, within the training sample and within the external testing sample. Analysis was conducted on carer-patient dyads where there were complete data across all relevant covariates.

### Testing predictive models

We first assessed the accuracy of the four predictive models in terms of how well the models predicted the observed ΔH_C_ at the individual level. For each of the four models, we generated two sets of predictions, one for the training sample and one for the testing sample. We compared each pair of predictions against the observed ΔH_C_ in the training and testing sample. For each comparison, we calculated the correlation between the observed and predicted values, the range of prediction errors, the mean absolute prediction error, and the root mean squared prediction error.

The second investigation focused on whether the models accurately predicted the mean ΔH_C_ over 12 months for groups of carers. This investigation was conducted because ΔH_C_ would be aggregated in trials and economic evaluations of healthcare interventions, rather than used to make judgements about the likely health outcomes of specific carers. The mean health effect for a group of carers (or family members) can be estimated and summed with the mean health effect for patients to calculate an incremental cost-effectiveness ratio [61]. A framework for including the family spillovers within economic evaluation is presented elsewhere [17]. We assigned carers to sub-groups based on whether the patient’s condition improved or worsened over the 12 months. This was a pragmatic way of trying to generate sub-groups of carers that might be experiencing different positive or negative spillovers from changes in the underlying clinical condition of the patient. We assigned carers to one of three groups based on whether: (i) one or more patient after-effects had improved over the last 12 months; (ii) there was no change in patient after-effects over the last 12 months; (iii) one or more patient after-effects had worsened over the last 12 months. We classified cases where one (or more) after effect improved and one (or more) after effect worsened as ‘mixed change’ and excluded these from this analysis as a non-‘Pareto’ change [54]. To predict the mean ΔH_C,_ we averaged the individual predictions of ΔH_C_ within each of the subgroups of carers. We assessed the accuracy of predictions at the group level by comparing the mean of the observations of ΔH_C_, with the mean of the predictions of ΔH_C_.

## Results

### Data

The response to the baseline survey on the family impact of meningitis is reported elsewhere [5]. When we followed up respondents 12 months later, we received 1038 responses. A total of 14 (1%) individuals declined to participate and 3 individuals were identified as ineligible prior to questionnaires being returned. During the process of data entry and cleaning, an additional 16 individuals were deemed ineligible, resulting in 1022 useable responses (net response rate 64%).

The final sample included 497 carer-patient dyads (Table 1) after exclusion of respondents not relevant to the predictive modelling. At baseline, the mean age of patients was 25 years old (SD 17). Around half of patients had some problems with anxiety or depression and over one-third had some limitations in usual activities and pain/discomfort. Carers also frequently reported limitations in their own health status, with over one-third reporting some anxiety or depression and over one-third reporting some pain or discomfort. Just over three-quarters of carers were parents of the patient. Only a minority of carers provided daily informal care for the patient.

The mean health status of both carers and patients deteriorated over the 12 months (Table 2). This deterioration on average in the health status of these two groups masks a wide range of improvements and deteriorations at the individual level. Specifically, 23% of carers and 24% of patients experienced an improvement in health status over the 12 months, and 36% of carers and 38% of patients experienced deterioration.

**Table 2:** Characteristics of patients and carers in the predictive modelling sample.

	Baseline	Follow-up
	(n = 497)	(n = 497)
**Patient**		
*Health status†*		
EQ-5D-5L (mean, SD)	0.79 (0.28)	0.77 (0.28)
Mobility problems (%)	23%	26%
Self-care problems (%)	19%	20%
Usual activities problems (%)	38%	40%
Pain problems (%)	35%	40%
Anxiety/depression problems (%)	45%	53%
*Meningitis-related after-effects‡*		
Behavioural/emotional problems	41%	28% improved and 37% worse
Mild-moderate learning disability	23%	16% improved and 25% worse
Scarring	20%	23% improved and 13% worse
Balance problems	18%	13% improved and 19% worse
Speech or language impairment	17%	25% improved and 16% worse
*Healthcare use in last 12 months*		
GP		68%
Hospital (outpatient)		50%
Hospital (inpatient)		15%
Age (mean, SD)	24.9 (17.3)	25.9 (17.3)
Sex (% female)	46%	46%
**Carer**		
*Health status*		
EQ-5D-5L (mean, SD)	0.86 (0.18)	0.84 (0.20)
Mobility problems (%)	16%	17%
Self-care problems (%)	4%	4%
Usual activities problems (%)	17%	18%
Pain problems (%)	36%	41%
Anxiety/depression problems (%)	42%	48%
Age (mean, SD)	51.9 (11.7)	52.9 (11.7)
Sex (% female)	88%	88%
*Relation to survivor*		
Parent (%)	78%	78%
Partner (%)	10%	10%
Grandparent (%)	7%	7%
Other (%)	5%	5%
Co-resident (yes)	64%	60%
Provide daily informal care (yes)	20%	25%

†‘Problems’ in each EQ-5D-5L item refer to a response of level 2 or lower.

‡The follow-up questionnaire included questions about whether the severity of after-effects had changed, rather than whether or not they were present. The second column reports the proportion of those with after-effect who experienced an improvement/no change/worsening over the 12 months.

**Fig 2:**
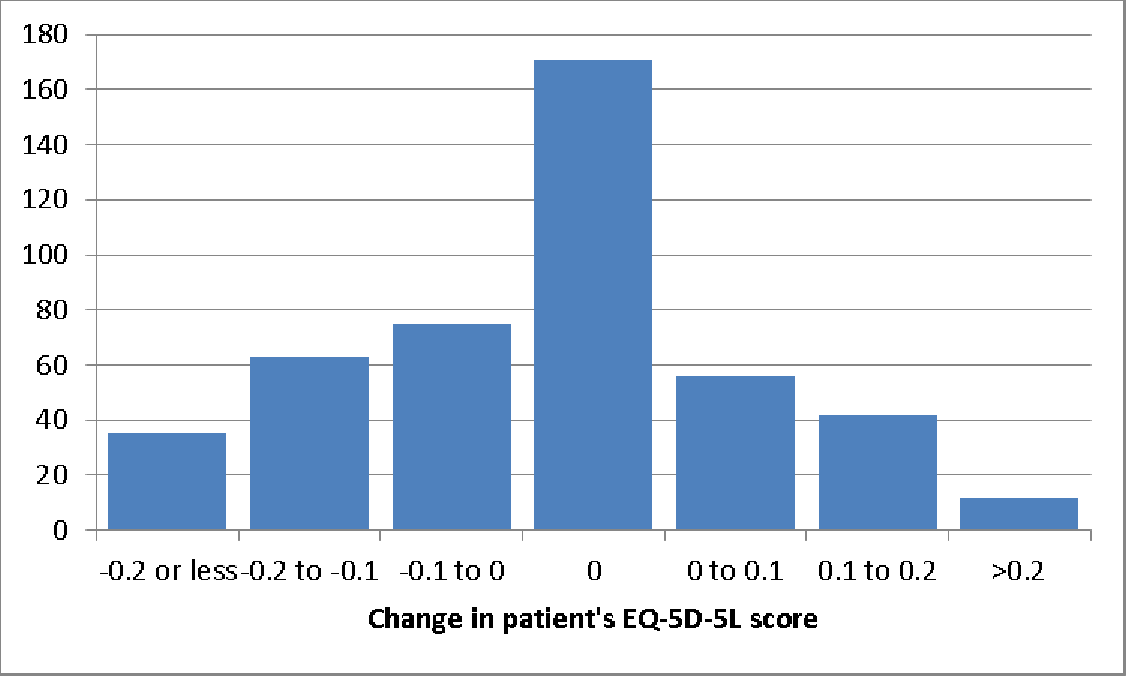
Distribution of change in patient’s EQ-5D-5L scores (n = 454).

**Fig 3:**
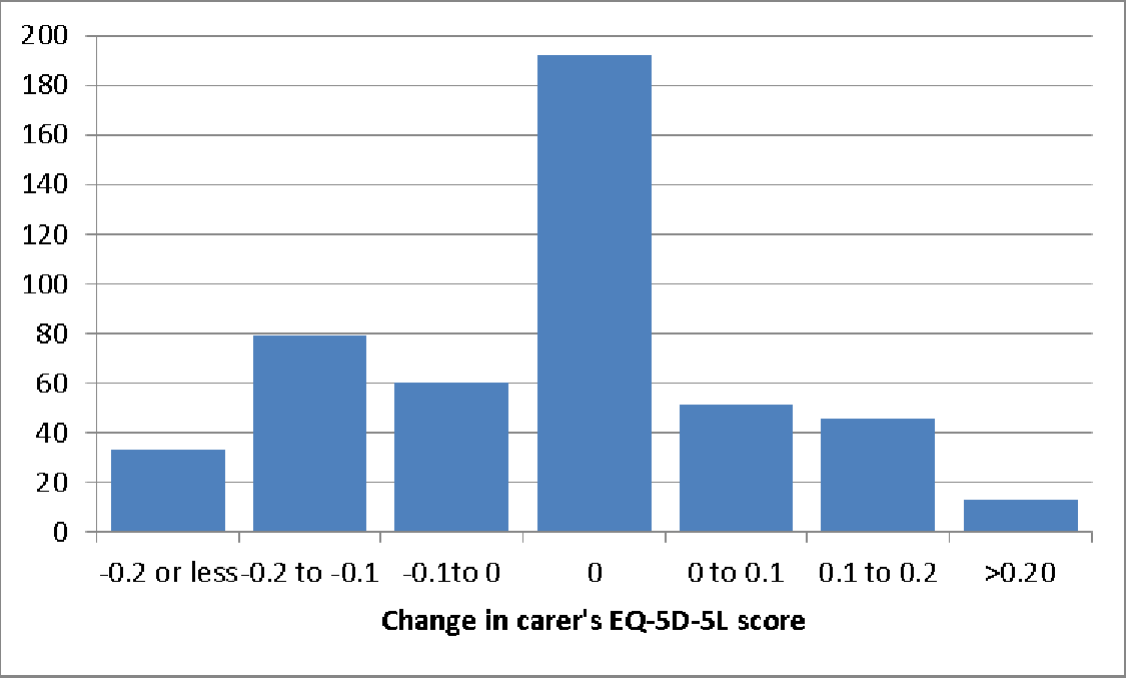
Distribution of change in carer’s EQ-5D-5L score (n = 474).

Figs 2 and 3 show the distribution of changes in carers’ and patients’ EQ-5D-5L scores. While a majority of carers and patients experienced some change in health status over the 12 months, large numbers had no reported change (hence the peaks at 0). Improvements in carer health status were more likely to occur when patient health status also improved (p < 0.01) suggesting some underlying basis for predicting ΔH_C_ using patient health status.

### Models to predict changes in carers’ EQ-5D-5L scores

Of the 497 carer-patient dyads, 249 were randomised to the training sample, and 248 were randomised to the testing sample. There were no significant differences (p<0.05) between carers and patients in the two samples, in terms of their health status, age or sex.

Table 3 shows the estimation of the four models to predict changes in carers’ EQ-5D-5L scores over 12 months. In model 1, the signs on the coefficients for improvements and deteriorations in aspects of the patient’s health status are mostly in the expected direction. For example a decline in patient mobility is associated with *negative* ΔH_C_ and an improvement in patient mobility with *positive* ΔH_C._ However there were anomalies, albeit statistically non-significant ones, such as the association between improvements in patient anxiety and depression and *negative* ΔH_C_. In model 2, only one of the additional five variables representing patient after-effects was significant at p<0.05. This was the presence of balance problems at baseline which was associated with a greater decline in carer health status. In model 3, the patient’s GP and inpatient care use over the last 12 months was associated with increased carer health status and the patient’s outpatient care use being associated with decreased carer health status. In model 4, the transitions into daily care and living with the care recipient were associated with positive ΔH_C_ as a biological relationship between patient and carer, although the latter was the only association significant at p<0.05.

**Table 3:** Models to predict carer EQ-5D-5L changes (ΔH_C_) from patient data.

Variables	MODEL 1	MODEL 2	MODEL 3	MODEL 4
Constant	-0.019 (0.31)	-0.026 (0.22)	-0.024 (0.34)	0.036 (0.48)
PATIENT DEMOGRAPHICS				
Age (years)	0.000 (0.72)	0.000 (0.33)	-0.000 (0.55)	-0.001 (0.26)
Sex (male)	-0.021 (0.22)	-0.021 (0.21)	-0.014 (0.43)	-0.013 (0.45)
PATIENT HEALTH STATUS CHANGE				
Mobility worsening	-0.091 (0.01)	-0.087 (0.02)	-0.084 (0.02)	-0.070 (0.06)
Mobility improvement	0.105 (0.01)	0.117 (0.01)	0.146 (0.00)	0.143 (0.00)
Self care worsening	0.026 (0.52)	-0.000 (0.99)	-0.007 (0.86)	-0.002 (0.95)
Self care improvement	0.007 (0.86)	0.014 (0.71)	0.020 (0.60)	0.024 (0.54)
Usual activities worsening	0.043 (0.07)	0.059 (0.15)	0.056 (0.02)	0.054 (0.03)
Usual activities improvement	0.060 (0.04)	0.070 (0.15)	0.062 (0.03)	0.072 (0.01)
Pain/discomfort worsening	-0.002 (0.92)	-0.001 (0.98)	-0.005 (0.84)	0.001 (0.98)
Pain/discomfort improvement	-0.012 (0.72)	-0.022 (0.50)	-0.014 (0.67)	-0.012 (0.72)
Anxiety/depression worsening	-0.039 (0.08)	-0.033 (0.12)	-0.035 (0.010)	-0.037 (0.08)
Anxiety/depression improvement	-0.044 (0.12)	-0.040 (0.15)	-0.043 (0.12)	-0.040 (0.15)
PATIENT AFTER-EFFECTS PRESENT				
Behaviour		0.017 (0.37)	0.015 (0.38)	0.014 (0.43)
Learning disabilities		-0.026 (0.21)	-0.025 (0.23)	-0.022 (0.31)
Scarring		0.015 (0.48)	0.009 (0.64)	0.010 (0.62)
Balance		-0.080 (0.02)	-0.081 (0.00)	-0.086(0.00)
Speech/language		0.022 (0.42)	0.011 (0.67)	0.007 (0.80)
PATIENT HEALTHCARE (12 MONTHS)				
GP			0.043 (0.02)	0.042 (0.03)
Outpatient			-0.033 (0.10)	-0.024 (0.23)
Inpatient			0.051 (0.10)	0.042 (0.18)
CARE-RELATED VARIABLES				
Age (years)				-0.001 (0.39)
Sex (male)				-0.015 (0.57)
Biological relationship (Yes)				0.084 (0.03)
Co-resident (No)				0.032 (0.16)
Daily care (Yes)				-0.005(0.83)
MODEL CHARACTERISTICS				
Observations	206	206	206	206
F	2.64	2.63	2.71	2.42
R^2^	0.141	0.192	0.227	0.251
Adj R^2^	0.088	0.119	0.143	0.147
AIC	-289	-291	-294	-291

### Predictive accuracy of models–individual level

The predictive accuracy of the four models is summarised in Table 4. Plots of the observed and predicted values indicated a tendency for improvements in carer health status to be under-predicted by the models and deteriorations to be over-predicted. Table 4 shows medium to strong correlations [62] between predicted and observed values in the training sample. However, the table also shows, for all models, the correlations between predicted and actual ΔH_C_ in the testing sample were negligible and non-significant. In the testing sample, the MAE and RMSE also increased with the addition of extra variables (i.e. from model 1 through to 4).

**Table 4:** Predictive accuracy of models of changes in carers’ health status.

	Correlation between observed and predicted ΔH_C_ (p value)	Range of absolute errors	MAE	RMSE	n
**MODEL 1**					
Training sample	0.38 (0.00)	0.001 to 0.471	0.079	0.116	206
Testing sample	0.02 (0.78)	0.001 to 0.596	0.092	0.124	209
**MODEL 2**					
Training sample	0.44 (0.00)	0.000 to 0.408	0.077	0.114	206
Testing sample	0.03 (0.64)	0.000 to 0.622	0.094	0.128	209
**MODEL 3**					
Training sample	0.48 (0.00)	0.001 to 0.411	0.077	0.113	206
Testing sample	0.02 (0.81)	0.001 to 0.609	0.096	0.133	209
**MODEL 4**					
Training sample	0.50 (0.00)	0.001 to 0.414	0.076	0.113	206
Testing sample	0.02 (0.75)	0.001 to 0.623	0.097	0.132	209

### Predictive accuracy of models–group level

As noted in 3.4, we generated three groups of carer-patient dyads to examine predictive accuracy at the aggregate level. Eighty (19%) carer-patient dyads experienced one or more after-effects improving and were allocated to the first sub-group. Two-hundred and eight (50%) carer-patient dyads experienced no change in after-effects and were allocated to the second sub-group. Ninety-two (22%) carer-patient dyads experienced one or more after-effects worsening and were allocated to the third sub-group. Finally 35 (8%) carer–patient dyads experienced mixed change in after-effects and were excluded from this element of the analysis. Table 5 summarises the mean observed and predicted ΔH_C_ for the three sub-groups. In this analysis, the predictions came from model 3; this was the model that used patient data that performed best in terms of explanatory power and predictions within the training sample. Table 5 indicates that the difference between the mean predicted changes and mean observed changes ranges from 0.002 to 0.020 across the six sub-groups.

**Table 5:** Observed and predicted mean ΔH_C_ in sub-groups of carer-patient dyads with full data (n = 415).

		Observed ΔH_C_	Predicted ΔH_C_
**One or more patient after-effect improved**	In sample (n = 42)	-0.051	-0.040
	Out of sample (n = 38)	-0.021	-0.023
**No change in any patient after-effects**	In sample (n = 106)	-0.020	-0.022
	Out of sample (n = 102)	-0.008	-0.028
**One or more patient after-effect worsened**	In sample (n = 38)	-0.019	-0.022
	Out of sample (n = 54)	-0.035	-0.040

## Discussion

The aim of this study was to investigate whether it was possible to use patient data to predict changes in the health status of family carers. We found that it was feasible to estimate models to predict changes in carers’ health status. However the predictions generated were poorly correlated with the observed changes in carers’ health status in an external testing sample.

The predictor variables were included on theoretical grounds, to cover the factors that affect the carer’s health status that might be collected routinely in clinical studies. In our regression models most of the predictor variables were found to be non-significant and these variables sometimes had counterintuitive signs. In terms of the patient’s health status, changes in mobility appeared to be the most important predictor of ΔH_C_. The positive impact of improved patient mobility on carer health status is consistent with the broader literature showing a positive association between carer and patient health status [12,63]. However the apparent prominence of patient mobility seems to conflict with other studies that have suggested that the patient’s mental health status or ability to self-care are more important predictors of carer outcomes [57,64].

We developed an additional predictive model using variables related to the carer, alongside patient variables, to assist in predicting ΔH_C_. The inclusion of care-related variables did improve the explanatory power of the model and led to slightly better predictions within the training sample. Clearly, accurate prediction of carer effects at an individual level requires more than just information about the patient’s health state. However, importantly, the additional variables did not improve predictions in the testing sample. This finding highlights the importance of using an external testing sample in predictive modelling. In the absence of an external testing sample it would be tempting to conclude that the more complex predictive models performed well, based on their greater explanatory power and predictive performance in sample. We also explored whether the ΔH_C_ for groups of carers could be predicted, as mean changes in health status for groups of the population are the focus of economic evaluation. This analysis suggested that predictive models may provide an indication of the magnitude of ΔH_C_ for groups of carers. Predictions are likely to be more favourable when examined at a group level, because the various environmental influences on carers’ health will tend to cancel out. Nevertheless the sub-group findings offer some encouragement for researchers who may wish to generate predictions of mean health effects for carers at a group level in other settings.

The most likely reason for low predictive accuracy at the individual level lies in the nature of the relationship between carer health status and patient health status. The findings in this study are consistent with Wittenberg and Prosser’s (2013) assertion that patient illness has a small detrimental impact on carer health that is highly context dependent. The limited ability to predict ΔH_C_ from patient variables is probably a sign that patient-related variables are one of many factors likely to influence ΔH_C_. A carer’s health status for example is also likely to be related to their own age and sex [36,65], their particular caring role, the amount of care provided [35,39], as well as various genetic and environmental factors [46], and even the country they live in [66].

We did try to control for some of these additional variables and this did not improve our ability to make out-of-sample predictions in the testing sample. This may be to be due to the large number of predictor variables with relatively unstable relationships with ΔH_C_. This means that coefficients that were optimised for use in the training sample may have been poorly suited to the testing sample. Furthermore, the small sample size was likely to be an issue. Splitting the dataset into training and testing samples halved the sample size and some of the variables had a low proportion of observations in certain categories.

Despite the findings there are important lessons for future research and practice. First, it seems very unlikely that a ‘one-size fits all’ model for predicting carer health status from patient variables is possible. We found that the patient EQ-5D limitations that most impacted on carers’ health status appeared to differ compared to what has been found in other studies [57,64]. We also found that predictive performance of models in ostensibly similar samples was very different. Second, it remains important to develop methods to better include carer outcomes in economic evaluation. Our findings suggest that further research in this area might be better focused on methods to collect data on carers’ outcomes, rather than to predict those outcomes. This may require identifying the key contexts in which carer data ought to be collected and developing methods for reaching carers and collecting a brief dataset that can inform the subsequent economic evaluation. Developing better methods for estimating carer and family effects has been highlighted as an important area of future research by the 2^nd^ US Panel on Cost-effectiveness [3].

In conclusion, predictive modelling of carer health status from patient data is attractive in theory, but appears to be highly problematic in practice. Our findings show that–in the context of long term afters of meningitis—predictive models can be estimated but performed poorly in an external testing sample. These findings appear to be due to the highly-context dependent relationship between carer and patient health status. These findings should not detract from the pressing need to properly include carer outcomes in economic evaluation. In view of this, efforts in research and practice efforts ought to focus on collecting carer data more routinely to enable carer outcomes to be properly reflected in economic evaluation.

## Supporting information

S1 File Comparison of the results of the three estimation techniques used for the four predictive models of carer health effects. (DOCX)
