# Supplementary material for: Predicting carer health effects for use in economic evaluation
Source: PLoS One. 2017 Sep 26;12(9):e0184886. doi: 10.1371/journal.pone.0184886 (PMC5614532; doi:10.1371/journal.pone.0184886)
Supplement: S1 File — (DOCX) [file pone.0184886.s001.docx]

**Comparison of estimation techniques for model 1**

| **Variables** | **OLS** | **ALDVMM** | **BETA** |
| --- | --- | --- | --- |
| Constant  PATIENT DEMOGRAPHICS  Age (years)  Sex (male)  PATIENT HEALTH STATUS CHANGE  Mobility Worse  Improvement  Self-care Worse  Improvement  Usual activities Worse  Improvement    Pain/discomfort Worse  Improvement  Anxiety/depression Worse  Improvement | 0.486 (0.00)  0.000 (0.72)  -0.021 (0.22)  -0.065 (0.01) 0.075 (0.01)  0.018 (0.52)  0.005 (0.86)  0.031 (0.07)  0.043 (0.04)  -0.002 (0.92)  -0.008 (0.72)  -0.028 (0.08)  -0.031 (0.12) | 0.486 (0.00)  0.000 (0.33)  -0.014 (0.21)  -0.065 (0.01) 0.075 (0.01)  0.018 (0.50)  0.005 (0.86)  0.031 (0.06)  0.043 (0.03)  -0.001 (0.92)  -0.008 (0.72)  -0.028 (0.07)  -0.031 (0.10) | -0.047 (0.39)  -0.000 (0.55)  -0.014 (0.43)  -0.272 (0.01) 0.381 (0.00)  0.078 (0.50)  -0.003 (0.98)  0.119 (0.09)  0.200 (0.02)  0.014 (0.82)  0.036 (0.72)  -0.112 (0.07)  -0.135 (0.10) |
| MODEL CHARACTERISTICS  AIC  BIC | -427  -384 | -425  -379 | -407  -360 |

**Comparison of estimation techniques for model 2**

| **Variables** | **OLS** | **ALDVMM** | **BETA** |
| --- | --- | --- | --- |
| Constant  PATIENT DEMOGRAPHICS  Age (years)  Sex (male)  PATIENT HEALTH STATUS CHANGE  Mobility Worse  Improvement  Self-care Worse  Improvement  Usual activities Worse  Improvement    Pain/discomfort Worse  Improvement  Anxiety/depression Worse  Improvement    PATIENT AFTER EFFECTS  Behavioural problems  Mild learning disability  Scarring  Balance problems  Speech problems | 0.482 (0.00)  0.000 (0.33)  -0.015 (0.21)  -0.062 (0.02) 0.083 (0.01)  -0.000 (0.99)  0.010 (0.71)  0.042 (0.02)  0.050 (0.02)  -0.000 (0.98)  -0.016 (0.50)  -0.024 (0.12)  -0.029 (0.15)  0.012 (0.37)  -0.018 (0.21)  0.010 (0.48)  -0.057 (0.00)  0.016 (0.42) | 0.482 (0.00)  0.000 (0.33)  -0.015 (0.21)  -0.062 (0.01) 0.083 (0.00)  -0.000 (0.99)  0.010 (0.70)  0.042 (0.01)  0.050 (0.01)  -0.000 (0.98)  -0.016 (0.48)  -0.024 (0.10)  -0.029 (0.13)  0.012 (0.34)  -0.018 (0.19)  0.010 (0.46)  -0.057 (0.00)  0.016 (0.40) | -0.0069 (0.25)  0.001 (0.33)  -0.074 (0.12)  -0.262 (0.01) 0.398 (0.00)  -0.013 (0.91)  0.023 (0.84)  0.172 (0.01)  0.230 (0.01)  0.018 (0.78)  -0.073 (0.44)  -0.096 (0.12)  -0.125 (0.12)  0.058 (0.26)  0.089 (0.13)  0.036 (0.54)  -0.252 (0.00)  0.090 (0.24) |
| MODEL CHARACTERISTICS  AIC  BIC | -430  -370 | -428  -365 | -412  -348 |

**Comparison of estimation techniques for model 3**

| **Variables** | **OLS** | **ALDVMM** | **BETA** |
| --- | --- | --- | --- |
| Constant  PATIENT DEMOGRAPHICS  Age (years)  Sex (male)  PATIENT HEALTH STATUS CHANGE  Mobility Worse  Improvement  Self-care Worse  Improvement  Usual activities Worse  Improvement    Pain/discomfort Worse  Improvement  Anxiety/depression Worse  Improvement    PATIENT AFTER EFFECTS  Behavioural problems  Mild learning disability  Scarring  Balance problems  Speech problems  PATIENT HEALTHCARE (LAST 12 MONTHS)  GP  Outpatient  Inpatient | 0.476 (0.00)  0.000 (0.98)  -0.008 (0.53)  -0.060 (0.02) 0.104 (0.00)  -0.005 (0.86)  0.015 (0.60)  0.040 (0.02)  0.045 (0.03)  -0.003 (0.84)  -0.010 (0.67)  -0.025 (0.10)  -0.031 (0.12)  0.011 (0.38)  -0.018 (0.23)  0.007 (0.64)  -0.058 (0.00)  0.008 (0.67)  0.031 (0.02)  -0.023 (0.10)  0.037 (0.10) | 0.476 (0.00)  0.000 (0.99)  -0.008 (0.51)  -0.060 (0.01) 0.104 (0.00)  -0.005 (0.86)  0.015 (0.57)  0.040 (0.01)  0.045 (0.02)  -0.003 (0.83)  -0.010 (0.66)  -0.025 (0.08)  -0.031 (0.10)  0.011 (0.36)  -0.018 (0.20)  0.007 (0.62)  -0.058 (0.00)  0.008 (0.66)  0.031 (0.02)  -0.023 (0.09)  0.037 (0.08) | -0.0069 (0.25)  0.000 (0.99)  -0.045 (0.56)  -0.256 (0.01) 0.483 (0.00)  -0.032 (0.78)  0.040 (0.71)  0.165 (0.02)  0.208 (0.01)  0.008 (0.90)  -0.053 (0.58)  -0.098 (0.10)  -0.133 (0.09)  0.056 (0.27)  -0.088 (0.13)  0.021 (0.72)  -0.256 (0.00)  0.059 (0.44)  0.128 (0.02)  -0.097 (0.09)  0.138 (0.11) |
| MODEL CHARACTERISTICS  AIC  BIC | -433  -363 | -431  -357 | -412  -348 |

**Comparison of estimation techniques for model 4**

| **Variables** | **OLS** | **ALDVMM** | **BETA** |
| --- | --- | --- | --- |
| Constant  PATIENT DEMOGRAPHICS  Age (years)  Sex (male)  PATIENT HEALTH STATUS CHANGE  Mobility Worse  Improvement  Self-care Worse  Improvement  Usual activities Worse  Improvement    Pain/discomfort Worse  Improvement  Anxiety/depression Worse  Improvement    PATIENT AFTER EFFECTS  Behavioural problems  Mild learning disability  Scarring  Balance problems  Speech problems  PATIENT HEALTHCARE (LAST 12 MONTHS)  GP  Outpatient  Inpatient  CARE-RELATED VARIABLES  Age (years)  Sex (male)  Biological relationship (Yes)  Co-resident (No)  Daily care (Yes) | 0.508 (0.00)  -0.001 (0.26)  -0.010 (0.45)  -0.050 (0.06) 0.102 (0.00)  -0.002 (0.95)  0.017 (0.54)  0.039 (0.03)  0.051 (0.01)  0.001 (0.98)  -0.009 (0.72)  -0.027 (0.08)  -0.029 (0.15)  0.010 (0.43)  -0.015 (0.31)  0.007 (0.62)  -0.061 (0.00)  0.005 (0.80)  0.030 (0.03)  -0.017 (0.23)  0.030 (0.18)  -0.001 (0.39)  -0.011 (0.57)  0.060 (0.03)  0.023 (0.16)  -0.004 (0.83) | 0.508 (0.00)  -0.001 (0.23)  -0.010 (0.42)  -0.050 (0.04) 0.102 (0.00)  -0.002 (0.95)  0.017 (0.52)  0.039 (0.02)  0.051 (0.01)  0.001 (0.97)  -0.009 (0.70)  -0.027 (0.06)  -0.029 (0.12)  0.010 (0.40)  -0.015 (0.28)  0.007 (0.60)  -0.061 (0.00)  0.005 (0.79)  0.030 (0.02)  -0.017 (0.20)  0.030 (0.15)  -0.001 (0.36)  -0.011 (0.54)  0.060 (0.02)  0.023 (0.13)  -0.004 (0.82) | 0.028 (0.84)  -0.003 (0.23)  -0.052 (0.30)  -0.211 (0.04) 0.478 (0.00)  -0.032 (0.88)  0.040 (0.60)  0.163 (0.02)  0.234 (0.00)  0.021 (0.74)  -0.047 (0.62)  -0.108 (0.07)  -0.123 (0.12)  0.052 (0.30)  -0.080 (0.18)  0.023 (0.69)  -0.267 (0.00)  0.053 (0.50)  0.127 (0.02)  -0.072 (0.21)  0.111 (0.21)  -0.002 (0.45)  -0.049 (0.50)  0.244 (0.02)  0.085 (0.18)  -0.034 (0.62) |
| MODEL CHARACTERISTICS  AIC  BIC | -430  -343 | -428  -338 | -411  -320 |
